# Exploring the Impact of Structure-Sensitivity Factors on Thermographic Properties of Dy^3+^-Doped Oxide Crystals

**DOI:** 10.3390/ma14092370

**Published:** 2021-05-02

**Authors:** Radosław Lisiecki, Jarosław Komar, Bogusław Macalik, Michał Głowacki, Marek Berkowski, Witold Ryba-Romanowski

**Affiliations:** 1 Institute of Low Temperature and Structure Research, Polish Academy of Sciences, ul. Okólna 2, 50-422 Wrocław, Poland; j.komar@intibs.pl (J.K.); b.macalik@intibs.pl (B.M.); w.ryba-romanowski@intibs.pl (W.R.-R.); 2 Institute of Physics, Polish Academy of Sciences, Al. Lotnikow 32/46, 02-668 Warsaw, Poland; glowacki@ifpan.edu.pl (M.G.); berko@ifpan.edu.pl (M.B.)

**Keywords:** luminescence, Dy-doped crystals, optical temperature sensors

## Abstract

Optical absorption spectra and luminescence spectra were recorded as a function of temperature between 295 K and 800 K for single crystal samples of Gd_2_SiO_5_:Dy^3+^, Lu_2_SiO_5_:Dy^3+^, LiNbO_3_:Dy^3+^, and Gd_3_Ga_3_Al_2_O_12_:Dy^3+^ fabricated by the Czochralski method and of YAl_3_(BO_3_)_4_:Dy^3+^ fabricated by the top-seeded high temperature solution method. A thermally induced change of fluorescence intensity ratio (FIR) between the ^4^I_15/2_→ ^6^H_15/2_ and ^4^F_9/2_ → ^6^H_15/2_ emission bands of Dy^3+^ was inferred from experimental data. It was found that relative thermal sensitivities S_R_ at 350 K are higher for YAl_3_(BO_3_)_4_:Dy^3+^ and Lu_2_SiO_5_:Dy^3+^than those for the remaining systems studied. Based on detailed examination of the structural peculiarities of the crystals it was ascertained that the observed difference between thermosensitive features cannot be attributed directly to the dissimilarity of structural factors consisting of the geometry and symmetry of Dy^3+^ sites, the number of non-equivalent Dy^3+^ sites, and the host anisotropy. Instead, it was found that a meaningful correlation between relative thermal sensitivity S_R_ and rates of radiative transitions of Dy^3+^ inferred from the Judd–Ofelt treatment exists. It was concluded that generalization based on the Judd–Ofelt parameters and luminescence branching ratio analysis may be useful during a preliminary assessment of thermosensitive properties of new phosphor materials.

## 1. Introduction

The remote temperature readout is a useful and meaningful method, and consequently, great attention has been addressed towards distinct advanced luminescence thermometers. For this purpose, various sophisticated luminescence systems and temperature sensor techniques have been proposed and elaborated on within the last decade. The luminescence sensors, in the form of lanthanide-doped optical systems, quantum dots, organic fluorophores, or biomolecules, may be applied as potential luminescence thermometers and their temperature-dependent spectroscopic peculiarities and sensing capabilities have been reported and compared in the comprehensive review papers, e.g., [1,2,3,4,5].

A temperature readout above a thousand degrees is possible for limited luminescence systems, but inorganic amorphous materials or lanthanide-doped crystals showing efficient emission within wide UV-Vis-NIR spectral regions can be satisfactorily utilized there, in contrast to fluorophores or bio-molecules, which are susceptible to destruction [6,7,8,9,10,11,12,13,14,15].

In the present work we deal with the thermosensitive properties of Dy^3+^-doped oxide crystals. Their advantage over other rare-earth-doped phosphors stems from a specific energy level scheme of the Dy^3+^ ions, in which the energy separation between the ^4^F_9/2_ luminescent level and the next lower-energy dysprosium excited state is considerable, approaching 7000 cm^−1^. As a consequence, the contribution of adverse multiphonon relaxation is substantially suppressed and the quantum efficiency of the ^4^F_9/2_ luminescence is significant and weakly affected by the temperature for the most optical materials doped with dysprosium when the luminescence admixture concentration is adequately restricted [16]. With respect to the structural and optical properties of the host, the visible emission of the dysprosium can be differently distributed within blue, green, and red spectral regions, and, consequently, these individual materials’ spectroscopic characteristics influence the resulting phosphor color. The diverse phosphor materials containing Dy^3+^ ions, e.g., single-doped with Dy^3+^ [17], double-doped with Dy + Mn [18], Dy + Eu [19], or triple-doped with Dy + Eu + Tb [20], have been described in numerous recent papers pointing out their utility for the design of novel lighting devices. The intensive development of UV and blue-emitting diode lasers, which can be applied as effective pumping sources, is extremely favorable for potential Dy^3+^-doped laser materials. Recent deficiency of these efficient excitation sources significantly affected the progress of visible solid state lasers utilizing dysprosium-doped crystals. Fortunately, this inconvenience has been recently overcome, and, for instance, the laser performance of YAG:Dy^3+^ garnet crystals has been documented [21], describing a 12% slope efficiency of visible laser operation that was attained by applying a GaN laser diode as the optical pumping source.

Furthermore, dysprosium-doped crystals and glasses can be considered as potential optical temperature sensors and, as a result, several papers have been devoted to verify these possibilities [22,23,24,25,26,27]. The majority of these works were devoted to the preparation and assessment of the thermographic performance of new materials. There are, however, recent papers reporting more in-depth considerations, including the analysis of structure-sensitive factors. E. Hertle et al. [28] have investigated temperature-dependent emission qualities of Dy^3+^ in YAG, YAP, YSO, YSZ, and CASO, examining the impact of the host, and Er^3+^-Pr^3+^ sensitizers’ incorporation. In another paper, E. Hertle et al. [29] reported the in-depth investigation of (Gd,Lu)AlO_3_:Dy^3+^ and (Gd,Lu)Al_5_O_12_:Dy^3+^, unraveling the effect of substituting Gd^3+^ by Lu^3+^ ions on the garnet structure durability and the spectroscopic features of these luminescent materials. Perera and Rabufetti [30] reported the investigation of the thermosensitive properties of polycrystalline NaLa_1‒x_Dy_x_(MO_4_)_2_ and Na_5_La_1‒x_Dy_x_(MO_4_)_4_ (M = Mo, W) materials, with special attention paid to the structural implication and the effect of Dy^3+^ concentration. All Dy^3+^-doped systems mentioned above were prepared in a polycrystalline form by a high temperature solid state reaction. This method of material synthesis is cheap and time saving. In this way, series of samples differing in the concentration of luminescent rare-earth ions or in the substitution of cations in the host structure can be fabricated easily.

Our work deals with the examination of single crystals of Gd_2_SiO_5_:5at.%Dy^3+^ (GSO), Lu_2_SiO_5_:5at.%Dy^3+^ (LSO), LiNbO_3_:1.94at.%Dy^3+^ (LNO), and Gd_3_Ga_3_Al_2_O_12_:1at.%Dy^3+^ (GGAG) fabricated by the Czochralski method, and of YAl_3_(BO_3_)_4_:4at.%Dy^3+^ (YAB) fabricated by the top-seeded high temperature solution method. The choice of the crystals represents a trade-off between an intention to gather a set of samples showing inherent structural dissimilarity on one hand, and the availability of samples with the highest possible quality to warrant reliability of results on the other hand. The samples listed above comply with these requirements. The technology of their crystal growth has been mastered previously during works aiming at the design of visible lasers [31,32,33,34,35]. It will be shown in the following that the availability of single crystal samples is very relevant for comprehensive and reliable spectroscopic study. The intention of our investigation is to determine thermosensitive properties not yet reported for the systems studied and to correlate the obtained results with structural implications, attempting to establish a generalization regarding the effect of structure-sensitivity factors on luminescence thermometric qualities of Dy^3+^-doped oxide crystals.

## 2. Materials and Methods

A Varian 5E UV-Vis-NIR spectrophotometer (Agilent, 5301 Stevens Creek Blvd, Santa Clara, CA 95051, USA) was applied to record the optical absorption spectra and 0.1 nm instrumental spectral bandwidth was then established. To determine crystal field splitting of Dy^3+^ excited multiplets, the absorption spectra were measured at a low temperature between 5 K and 10 K. For these low-temperature experiments, the crystals were mounted into an Oxford Model CF 1204 cryostat containing a liquid helium flow system and an adequate temperature controller. To record absorption spectra at different temperatures between 295 K and 800 K, the samples were placed into a chamber furnace. An Edinburgh Instruments FLS980 fluorescence spectrophotometer (Edinburgh Instruments Ltd. 2 Bain Square, Kirkton Campus, EH54 7DQ, UK) was utilized to measure the survey luminescence spectra and excitation spectra. A 450 W xenon lamp was utilized as an excitation source, and a Hamamatsu 928 PMT photomultiplier (Hamamatsu, 430-0852 2-25-7 Ryoke, Naka-ku, Japan) was used as the photon-sensitive detector. The acquired spectra were corrected on the experimental response of the used apparatus, employing their adequate sensitivity and spectral ranges. For measurements performed at a higher temperature, within 295–800 K, the samples were placed into a chamber furnace. The appropriate thermocouple was applied to temperature detection, and measurement accuracy was verified by a proportional-integral-derivative (PID) Omron E5CK controller. The samples were excited at 355 nm by a light beam consisting of a spectral band with 15 nm FWHM provided by the filtered output of a xenon lamp. The emission spectra were measured as a function of temperature within 295–800 K utilizing an Optron DM711 monochromator (DongWoo Optron Co. Ltd., Kyungg-do, Korea) with a 750 mm focal length. The resulting luminescence signal was detected applying a R3896 photomultiplier (Hamamatsu, 430-0852 2-25-7 Ryoke, Naka-ku, Japan).

## 3. Results and Discussion

**Figure 1:**
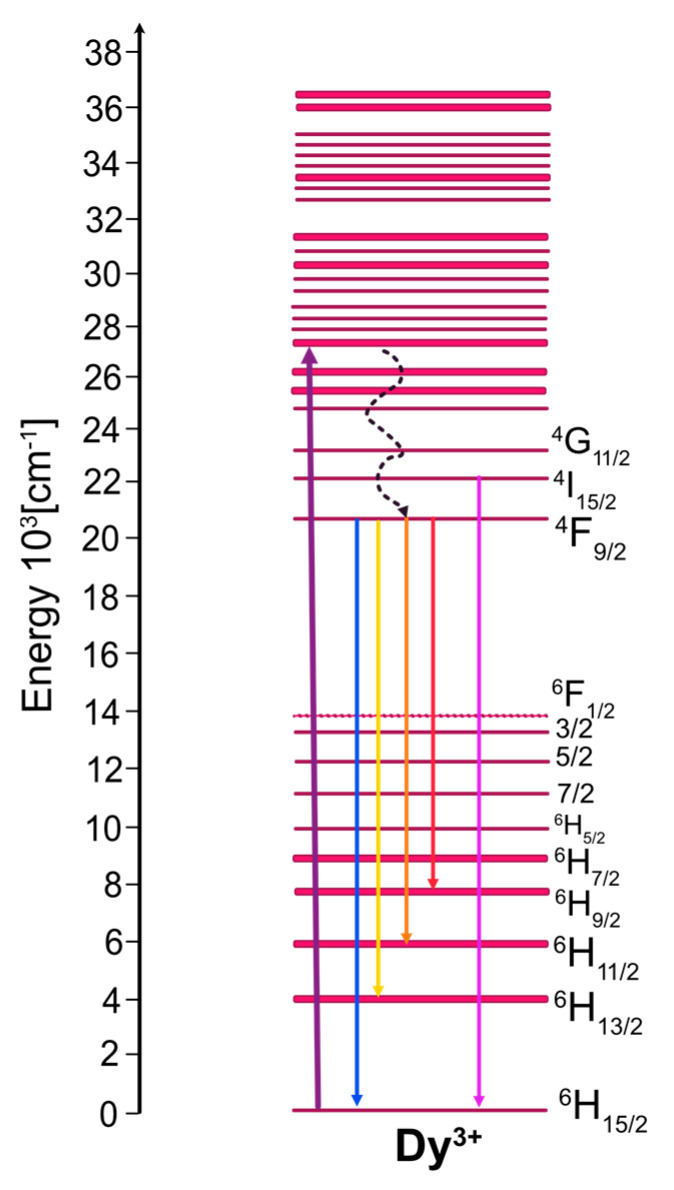
Energy levels scheme of Dy^3+^.

Experimental data will be interpreted referring to the fundamental structural and optical data of the host crystals gathered in Table 1 and the energy level scheme for Dy^3+^ depicted in Figure 1. To construct this figure, the energy values for excited states determined in the past for Dy^3+^ (aquo) were taken from [36].

**Figure 2:**
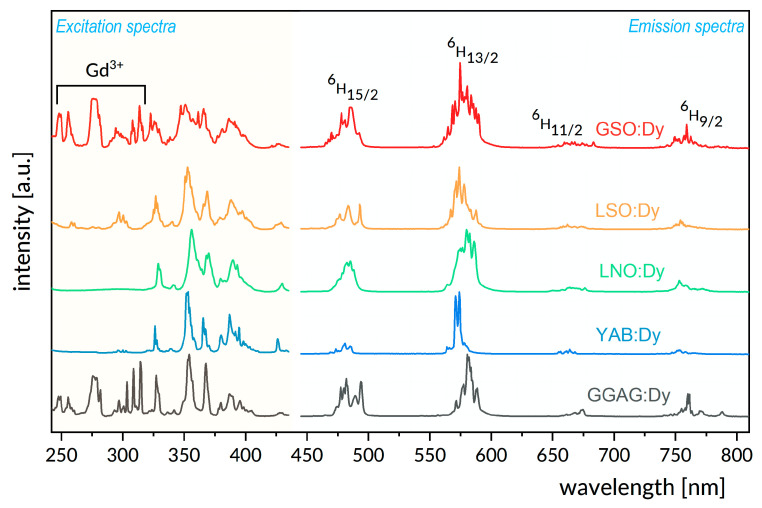
Survey spectra of visible luminescence (**right**) and survey excitation spectra of luminescence monitored at 575 nm (**left**) recorded at room temperature for the systems studied.

The levels involved in luminescent transitions considered here are labelled with the symbols ^2S+1^L_J_ of corresponding multiplets. Actually, for an ion imbedded in a crystalline host, each multiplet is split by the crystal field into crystal field components. Their number depends on the strength and symmetry of the crystal field, and, hence, on the structural features of the host crystal. In principle, low temperature absorption and luminescence spectra are able to offer detailed information regarding the number and nature of energy levels of rare-earth ions in crystals. Therefore, the interpretation of the observed luminescence phenomena refers to energy levels inferred from low temperature optical spectra for each Dy^3+^-doped system under study. It follows from Figure 1 that excited multiplets created by the spin orbit splitting of the sextet ^6^H and ^6^F terms form a group of low energy levels located below about 14,300 cm^−1^. A second group consists of high energy levels above about 20,400 cm^−1^, related to closely spaced multiplets derived from the ^4^F, ^4^G, ^4^H, ^4^I, ^4^K, ^4^L, and ^4^M quartet and ^6^P sextet terms. It is worth noticing here that the 4f^9^ configuration of Dy^3+^contains levels actually located at higher energies than those depicted in Figure 1. They have been omitted for the sake of clarity. Energy separation ΔE between neighboring excited levels of rare-earth ions in solids is a governing factor that determines the competition between radiative decay and nonradiative multiphonon relaxation. The latter process involves the simultaneous emission of the highest energy phonons available in the host, and the rate W_mph_ of this process depends on ΔE according to the energy gap law W_mph_ = Cexp(−α ΔE), where C and α are host-dependent parameters. In the crystals studied, the high energy excited levels of Dy^3+^ ions relax nonradiatively, feeding the ^4^F_9/2_ luminescent level. Its decay is governed by radiative transitions, mainly because the energy separation ΔE of ~7000 cm^−1^ between the ^4^F_9/2_ level and the lower energy ^6^F_1/2_ level is large when compared to the phonon energies listed in Table 1. The ^4^F_9/2_ luminescence is related to the radiative transitions that terminate on multiplets derived from the ^6^H and ^6^F sextet terms. Transitions in the visible region are assigned and indicated by solid downward arrows in Figure 1. Transitions to remaining terminal levels are in the near infrared region and their intensities are small when compared to those in the visible region for virtually all Dy^3+^-doped hosts. Figure 2 compares survey spectra of visible luminescence recorded at room temperature for the systems studied. The spectra shown deserve some comments to make the comparison meaningful. First, it follows from Table 1 that, except for cubic GGAG:Dy, the remaining crystals are anisotropic, i.e., GSO:Dy and LSO:Dy are optically biaxial whereas LNO:Dy and YAB:Dy are uniaxial. Their anisotropy was determined based on polarized optical spectra and has been reported in the past. Optical anisotropy is not relevant to our study; accordingly, the spectra in Figure 2 and all other spectra shown later on were recorded with unpolarized light. Second, instrumental spectral bandwidths for our measurement were carefully checked to avoid instrumental line broadening. With these points clarified, the impact of structural peculiarities listed in Table 1 on the spectral features of the luminescence bands becomes easier to see. Dy^3+^ ions substitute Gd^3+^ in GSO, and Lu^3+^ in LSO. They reside in two nonequivalent sites differing in the coordination number (CN), namely 9 and 7 for GSO [37] or 7 and 6 for LSO [38]. In the crystal structure of GSO, the two sites differ also in their local symmetry. Luminescence bands for GSO:Dy and LSO:Dy presented in Figure 2 show large overall widths and reach structures that stem from partly overlapping transitions between crystal field levels of two kinds of Dy^3+^ ions having dissimilar energies. In LNO, Dy^3+^ ions substitute in principle Li^+^ ions entering sites characterized by CN = 6 and local symmetry close to C_3_ [39]. However, observed spectra of LNO:Dy luminescence show large spectral width and poor band structure pointing at strong inhomogeneous broadening of spectral lines.

**Figure 3:**
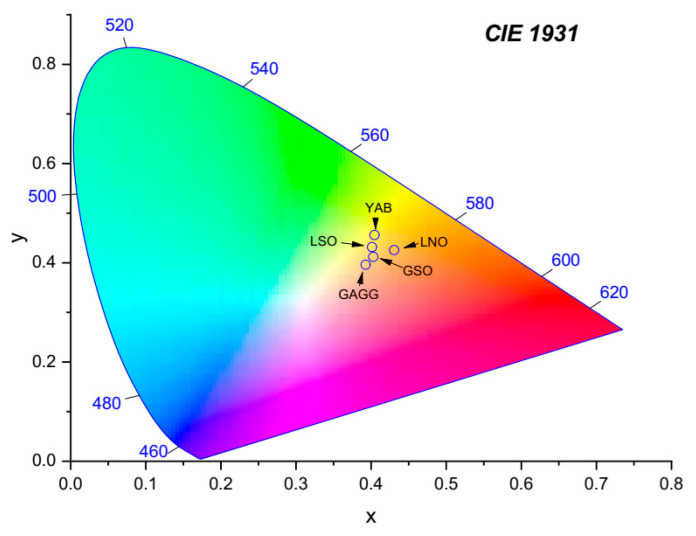
CIE chromacity diagram constructed based on the color coordinates gathered in the bottom of Table 2.

This effect is induced by the inherent structural disorder in a congruent LNO host, combined with problems with charge compensation in doped samples. In YAB, the Dy^3+^ ions substitute Y^3+^ ions entering one kind of well-defined site with CN = 6 and C_3_ local symmetry [40]. As a consequence, observed spectral bands are relatively narrow and show some structure. In GGAG the Dy^3+^ ions substitute Gd^3+^ ions entering sites with CN = 8 and D_2_ local symmetry [41]. GGAG host shows the structural disorder inherent for solid state solution crystals. Partial substitution of gallium ions by aluminum ions in this host brings about a dissimilarity of the crystal field acting on Dy^3+^ ions in different sites, inducing inhomogeneous spectral broadening, which, in contrast to LNO:Dy, is intentional. It can be seen in Figure 2 that the host crystal studied also affects the spectral distribution of luminescence intensity of incorporated Dy^3+^ ions, although the ^4^F_9/2_ → ^6^H_13/2_ band invariably dominates the spectra. Quantitative assessment of the distribution of luminescence intensity among spectral bands is commonly expressed in terms of luminescence branching ratios β, defined as the ratio of radiative transition rate for a particular transition from a luminescent level to the sum of rates of radiative transitions to all terminal levels. Experimental β_exp_ values can be evaluated by the numerical integration of bands in luminescence spectra. Table 2 compares percent values of β_exp_ determined by the numerical integration of spectra in Figure 2. It should be noticed that the sums of β_exp_ for four visible transitions equal to 100% because the contribution of weak infrared transition was neglected. Differences in the color of emitted light resulting from the dissimilarity of branching ratio values can be revealed based on the CIE chromacity diagram shown in Figure 3 and the color coordinates gathered in the lowest part of Table 2.

In excitation spectra shown in Figure 2, the complex structure of bands is due to transitions within the 4f^9^ configuration of incorporated Dy^3+^ ions, except for strong Gd^3+^ bands located at around 250 nm and 310 nm in GSO:Dy and GGAG:Dy. The band located between about 340 nm and 360 nm is the most prominent. Its high intensity is due essentially to the ^6^H_15/2_ → ^6^P_7/2_ transition, although those ending on (^4^P, ^4^D)_3/2_, ^6^P_5/2_, ^4^I_11/2_, (^4^M, ^4^I)_15/2_, (^4^F, ^4^D)_5/2_, and ^4^I_9/2_ levels are also involved. These spectra imply that the intensity of Dy^3+^ luminescence depends critically on the wavelength of the incident excitation light. This shortcoming may not be encountered at higher temperatures because of thermal effects. Optical absorption and emission spectra of rare-earth ions located in non-centrosymmetric sites are related to pure electric dipole transitions, except for ions from the beginning or the end of rare-earth series, which show the contribution of vibronic transitions.

**Figure 4:**
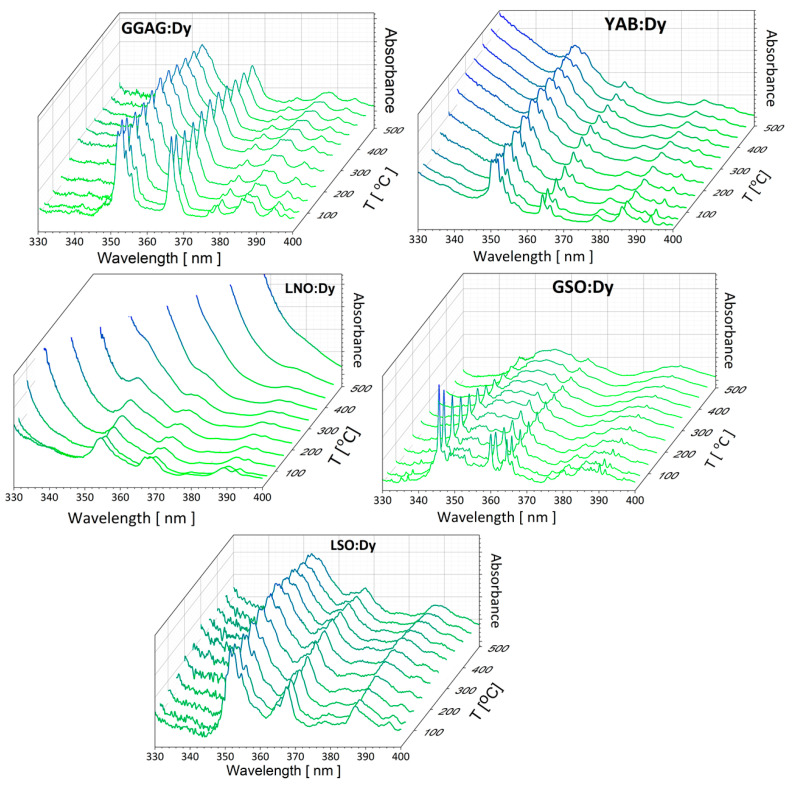
Optical absorption spectra in the UV-blue region recorded at several different temperatures between 295 K and 775 K for the systems under study. For the sake of clarity, the spectral region was restricted to 330–400 nm.

Thermally induced changes of the spectral bands of electric dipole transitions between multiplets of rare-earth ions in solids result from several factors. The governing factor follows from Boltzmann statistics, which determine the relative population of crystal field levels within multiplets, revealing, thereby, the number of band components and their intensity contribution as a function of the temperature. Other important factors relevant to narrow lines and related to transitions between individual crystal field levels are as follows: (i) thermal line broadening, a mechanism consisting of the Raman scattering of phonons by an ion in an excited state and (ii) thermal line shift, which determines the change of transition energy due to the temperature-induced displacement of levels involved in the transition. It is worth noticing here that the factors mentioned above affect the shapes of spectral bands and do not change the rates of the radiative transitions involved. Figure 4 compares optical absorption spectra in the UV-blue region recorded at several different temperatures between 300 K and 775 K for the systems under study. For the sake of clarity, the spectral region was restricted to 330–400 nm, where the most intense bands of interest for excitation purposes were located. In all spectra shown, the contribution of intense narrow lines and of local maxima diminishes with growing temperature, and eventually, above about 600 K, the spectra consist of a few broad and structureless bands. Spectra of GSO:Dy^3+^ provide a spectacular example of such an evolution, but the change of those for LSO:Dy^3+^ is less impressive. It follows from data in Table 1 that these orthosilicate hosts have ordered structures offering two different sites for Dy^3+^ ions. For each Dy^3+^ site, the crystal field splits the ^6^H_15/2_ ground multiplet into eight components. As a consequence, partly overlapping homogeneously broadened lines related to transitions from 16 initial crystal field components contribute to the absorption bands of LSO:Dy and GSO:Dy. Low temperature luminescence spectra provided the overall ground state splitting of 933 cm^−1^ for Dy1 and Dy2 sites in LSO [35]. The overall ground state splitting of 922 cm^−1^ for the low symmetry Dy2 site and of 598 cm^−1^ for the high symmetry Dy1 site have been determined for GSO [34]. Different site symmetries combined with different ground state splitting results in the dissimilarity of LSO:Dy and GSO:Dy absorption spectra observed at room temperature. It can be seen in Figure 4 that this dissimilarity disappears gradually with increasing temperature. This is due to the increasing contribution of lines from higher energy crystal field components of the initial multiplet combined with thermal line broadening and thermal line shift. Unlike LSO:Dy and GSO:Dy, the Dy^3+^ ions are located in one kind of sites in a disordered structure of GGAG. As a consequence, their absorption bands consist of a superposition of lines related to transitions from eight crystal field components of the ^6^H_15/2_ ground state, which shows an overall crystal field splitting of 674 cm^−1^ [31]. Owing to inhomogeneous line broadening, the spectral linewidths depend weakly on the temperature. Nevertheless, large inherent linewidths of several tens of nanometers combine with the increasing contribution of lines from higher energy crystal field components of the initial multiplet, contributing, thereby, to the thermally-induced broadening of the absorption bands. It is worth noticing that the spectra commented above do not contain bands of broad UV-blue absorption, indicating, thereby, that samples are free from point (color) defects. In the ordered structure of YAB, the Dy^3+^ ions substitute yttrium ions, and are located in one kind of site with CN equal to six and local symmetry D_3_. In principle, their absorption bands should consist of a superposition of narrow lines related to transitions from eight crystal field components of the ^6^H_15/2_ ground state, which shows an overall crystal field splitting of 468 cm^−1^ [32].

However, it can be seen in Figure 4 that, at 300 K, the baseline of the YAB:Dy spectra rises gently with decreasing wavelengths, but suffers from an upward shift at higher temperatures. This behavior indicates that the crystal structure of our YAB sample contains point defects, which show a thermally induced increase of absorption intensity. Occurrence of point defects gives rise to some inhomogeneous broadening of narrow band components, whereas a resulting parasitic absorption may adversely affect the efficiency of the Dy^3+^ excitation. It follows also from Figure 4 that these shortcomings are crucially relevant to the LNO:Dy system. Owing to a strong thermally induced increase of absorption intensity, which we interpret in terms of temperature-dependent charge transfer (CT) transition [42], the absorption bands of Dy^3+^ in the UV-blue region disappear in spectra recorded above about 500 K.

**Figure 5:**
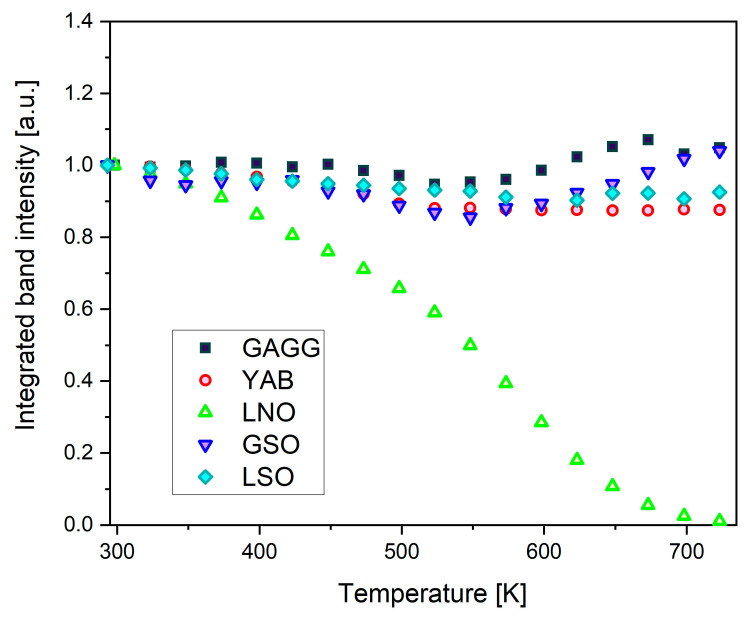
Integrated Dy^3+^ luminescence intensity within the 425–800 nm region at different temperatures between 295 K and 725 K. Results were normalized to unity at 295 K.

Recorded absorption spectra make it possible to determine quantitatively the effect of the sample temperature on Dy^3+^ luminescence intensity. For each system studied, the overall Dy^3+^ luminescence spectra recorded at different temperatures between 295 K and 725 K were numerically integrated within the 425–800 nm region. Next, the integrated luminescence intensities were normalized to unity at 295 K. Figure 5 compares the results obtained when exciting the samples at 355 nm with light consisting of a spectral band 15 nm FWHM provided by a filtered output of a xenon lamp. It can be seen in Figure 5 that, for the samples studied, the Dy^3+^ luminescence intensity excited at about 355 nm depends weakly on the temperature, except for the LNO:Dy crystal. It can be noticed also that, beginning at about 600 K, the YAB:Dy luminescence intensity is the lowest, likely because of the adverse contribution of defect centers commented on above.

In the following, we examine luminescence phenomena related to transitions from the ^4^I_15/2_, ^4^F_9/2_ excited levels that are separated by about 1000 cm^−1^, and whose populations are therefore governed by Boltzmann statistics. Accordingly, a thermally-induced change of fluorescence intensity ratio (FIR) between the ^4^I_15/2_ → ^6^H_15/2_ and ^4^F_9/2_ → ^6^H_15/2_ emission bands is a temperature-dependent parameter that can serve for temperature sensing. At 300 K, the Dy^3+^ luminescence spectrum consists essentially of the ^4^F_9/2_ → ^6^H_15/2_ band in the 465–500 nm region. With increasing temperature, the ^4^I_15/2_ emission intensity between 450 nm and 465 nm, grows at the expense of the ^4^F_9/2_ emission intensity.

Accordingly, the luminescence intensities are proportional to the population of the involved energy levels, and the FIR of two thermally coupled levels can be defined by the following equation [43]:

(1) $$\mathrm{FIR}=\frac{I_{\left(4I_{15/2}\right)}}{I{(}_{4F_{9/2})}}=B\exp\left(-\frac{\Delta E}{\mathrm{kT}}\right)$$

where B is the temperature-independent constant, ΔE is the energy gap between the two thermally coupled levels, and k is the Boltzmann constant. An optical thermometer may be quantitatively characterized with the absolute or relative thermal sensitivity. The former parameter reveals the absolute FIR change with temperature variation and is expressed as:

(2) $$S_{A}=\frac{\mathrm{dFIR}}{\mathrm{dT}}=\;\mathrm{FIR}\frac{\Delta E}{{\mathrm{kT}}^{2}}$$

To compare the thermometers’ quality, the relative sensitivity is usually used because this parameter determines the normalized change of FIR with temperature variation, and is defined as [44]:

(3) $$S_{R}=\frac{1}{\mathrm{FIR}}\frac{\mathrm{dFIR}}{\mathrm{dT}}\cdot 100\%\;=\frac{\Delta E}{{\mathrm{kT}}^{2}}\cdot 100\%$$

**Figure 6:**
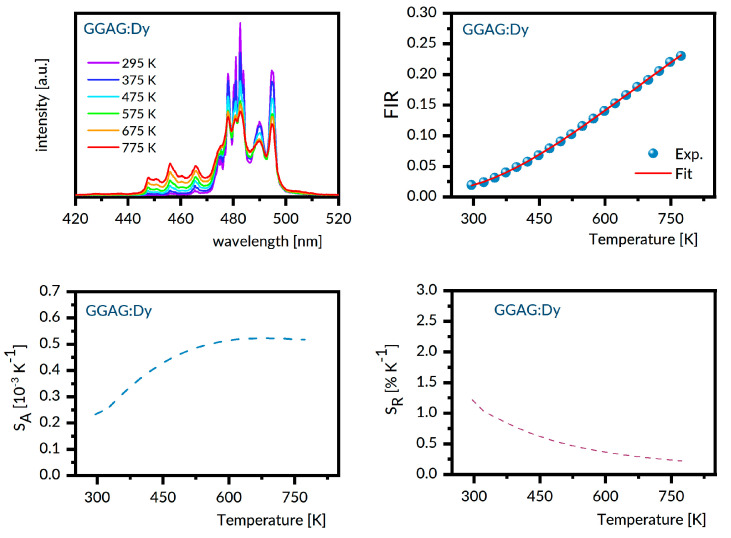
GGAG:Dy emission spectra recorded at several different temperatures (**upper left**), the plot of FIR versus temperature (**upper right**), the temperature dependence of S_A_ (**lower left**), and the temperature dependence of S_R_ (**lower right**).

**Figure 7:**
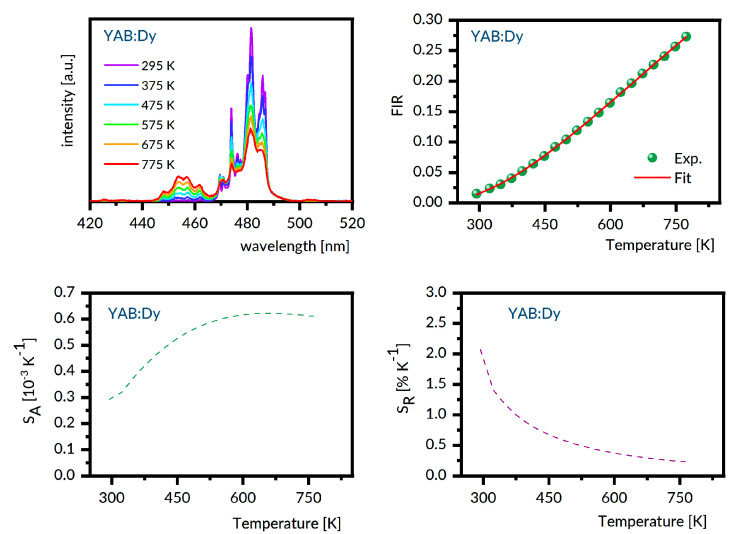
YAB:Dy emission spectra recorded at several different temperatures (**upper left**), the plot of FIR versus temperature (**upper right**), the temperature dependence of S_A_ (**lower left**), and the temperature dependence of S_R_ (**lower right**).

**Figure 8:**
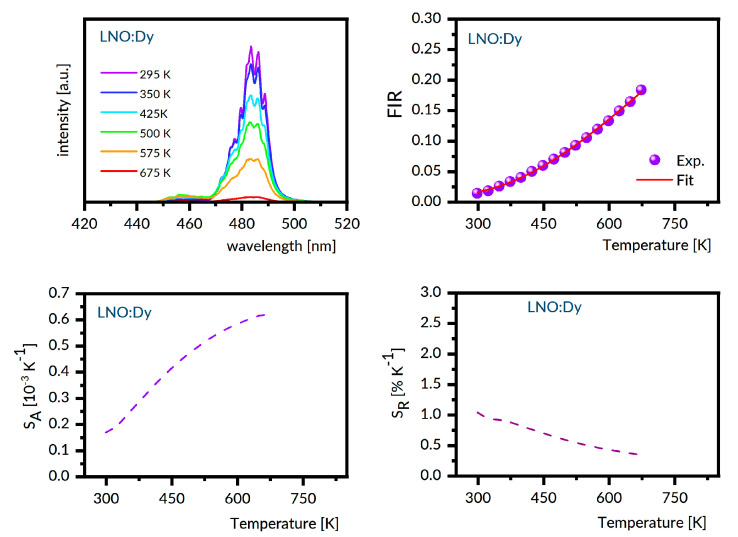
LNO:Dy emission spectra recorded at several different temperatures (**upper left**), the plot of FIR versus temperature (**upper right**), the temperature dependence of S_A_ (**lower left**), and the temperature dependence of S_R_ (**lower right**).

**Figure 9:**
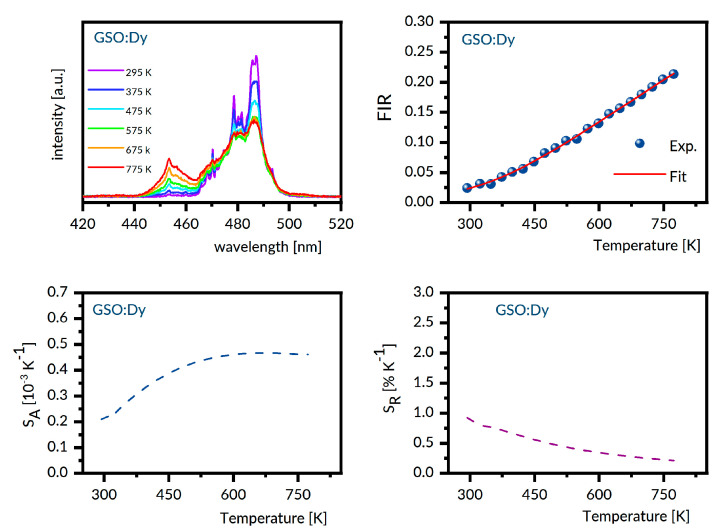
GSO:Dy emission spectra recorded at several different temperatures (**upper left**), the plot of FIR versus temperature (**upper right**), the temperature dependence of S_A_ (**lower left**), and the temperature dependence of S_R_ (**lower right**).

**Figure 10:**
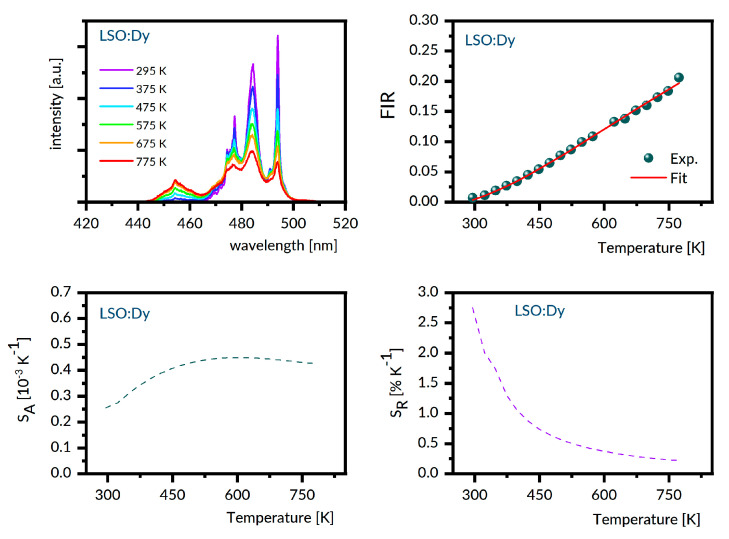
LSO:Dy emission spectra recorded at several different temperatures (**upper left)**, the plot of FIR versus temperature (**upper right**), the temperature dependence of S_A_ (**lower left**), and the temperature dependence of S_R_ (**lower right**).

For the samples under study, the luminescence spectra in the region 440–800 nm were recorded at different temperatures between 300 K and 800 K with steps of 25 K. Next, the experimental FIR values were evaluated by numerical integration of the recorded spectra. The best fit between the experimental temperature dependence of the FIR values and that predicted by Equation (1) provides the ΔE value involved. With these data, the S_A_ and S_R_ were determined as a function of the temperature from Equations (2) and (3), respectively. Figure 6, Figure 7, Figure 8, Figure 9 and Figure 10 present the results obtained.

**Figure 11:**
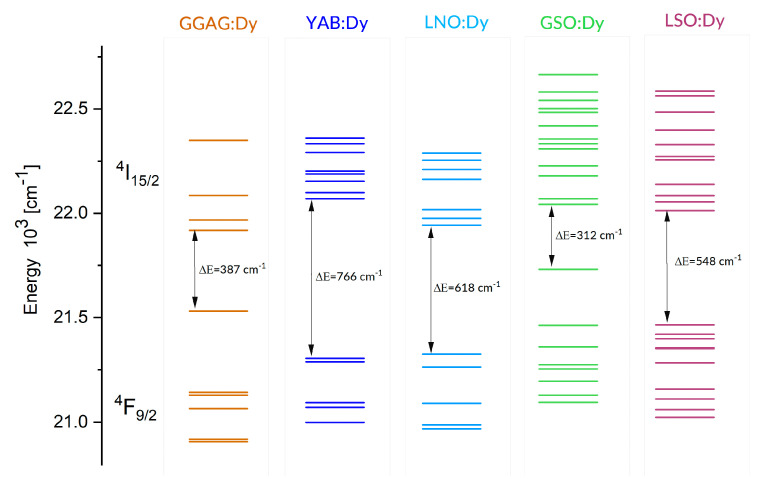
The crystal field splitting of the ^4^I_15/2_ and ^4^F_9/2_ multiplets of Dy^3+^, determined based on low temperature absorption spectra.

Each figure contains graphs showing spectra at several different temperatures (upper left), the plot of FIR versus temperature (upper right), the temperature dependence of S_A_ (lower left), and the temperature dependence of S_R_ (lower right). Our S_R_ values evaluated at 350 K and 750 K are compared in Table 3 to corresponding data reported for other Dy-doped crystals. It should be noted here that the luminescence intensity for LNO:Dy diminishes steeply with increasing temperature, restricting the reliability of the FIR and thermal sensitivity data at temperatures below 650 K. At this stage, the data in Table 3 deserve some comments. First, the S_R_ value at 350 K is greater than that at 750 K for all systems gathered, indicating that they are most suitable for near room temperature sensing. Second, the effect of temperature on S_R_ for different crystals is not the same. For instance, the change in temperature from 350 K to 750 K reduces the S_r_ by a factor of roughly seven for LSO:Dy and by a factor of three only for GSO:Dy. On the other hand, the change in crystal host is able to change the S_R_ values by no more than a factor of two, roughly. The ΔE values defined by Equation (1) and involved in plots of FIR versus temperature in Figure 6, Figure 7, Figure 8, Figure 9 and Figure 10 are given in the second column of Table 4 as ΔE_calc_ values. When discussing our results, we refer to the luminescence spectra presented in Figure 6, Figure 7, Figure 8, Figure 9 and Figure 10 and the energy level scheme in Figure 11 that was constructed based on the low temperature absorption spectra shown in References [31,32,33,34,35].

The energy differences ΔE_exp_ between centroids of the ^4^I_15/2_ → ^6^H_15/2_ and ^4^F_9/2_ → ^6^H_15/2_ emission bands at 350 K and 750 K were determined numerically and given in the second and third columns, respectively. The resulting ΔE_exp_ − ΔE_calc_ values appear in two end columns. It can be seen that, for all systems studied, the ΔE_calc_ and ΔE_exp_ values differ. Our results are consistent with those obtained recently by Perera and Rabufetti during their investigation of the thermosensitive properties of polycrystalline NaLa_1−x_Dy_x_(MO_4_)_2_ and Na_5_La_1−x_Dy_x_(MO_4_)_4_ (M = Mo, W). It has been observed that the calculated energy gaps ΔE_calc_ are systematically smaller than the experimental values ΔE_exp_ at 350 K, and this dissimilarity ranged from 95 cm^−1^ to 350 cm^−1^ [30].

The reasons for these dissimilarities are not obvious, deserving, therefore, a closer investigation. When discussing our results, we refer to the luminescence spectra presented in Figure 6, Figure 7, Figure 8, Figure 9 and Figure 10 and the energy level scheme in Figure 11 that was constructed based on low temperature absorption spectra. In principle, thermally induced changes of width and shape of the luminescence band related to the transition between multiplets of rare-earth ions in solids can be determined easily, provided the crystal field splitting of multiplets involved is known and the rates of transitions between individual crystal field levels are equal. Unfortunately, the latter condition is not always fulfilled in real systems. Hence, the former condition is not fulfilled frequently because a negligible intensity of some transitions prevents the location of levels involved.

Let us consider the LNO:Dy and LSO:Dy systems, which show the most significant disparity. It can be seen in Figure 11 that the ^4^F_9/2_ metastable multiplet of Dy^3+^ in LNO is split by the crystal field into five components, all of them located from low temperature absorption spectra. The higher-energy thermally coupled multiplet ^4^I_15/2_ is split by the crystal field into eight components, but only seven are located experimentally. The energy difference between the lowest component of the ^4^I_15/2_ multiplet and the highest component of the ^4^F_9/2_ multiplet is 618 cm^−1^. When the temperature grows, the population of the higher energy components increases at the expense of the lower energy components for the ^4^I_15/2_ and ^4^F_9/2_ excited multiplets and for the ground ^6^H_15/2_ multiplet. Anticipated changes of the luminescence bands consist of (a) a shift of the high energy wing towards shorter wavelengths and (b) an increase of intensity within the high energy wing due to a vanishing contribution of the self-absorption in this spectral region. It can be seen in Figure 8 that, in LNO:Dy, the anticipated changes are not corroborated by thermally induced changes of the experimental ^4^F_9/2_ → ^6^H_15/2_ luminescence band, which shows a nearly symmetric band-shape, weakly affected by the temperature. This may happen if thermally populated higher energy crystal field components of the initial ^4^F_9/2_ multiplet have small transition rates.

Markedly different luminescent features were observed for LSO:Dy. For each of two Dy^3+^ sites in this host, the ^4^F_9/2_ metastable multiplet is split by the crystal field into five components. In total, eight components were located from the low temperature absorption spectra. There are 16 components of the ^4^I_15/2_ multiplet, but only 11 were located experimentally. The energy difference between the lowest component of the ^4^I_15/2_ multiplet and the highest component of the ^4^F_9/2_ multiplet is 548 cm^−1^. It can be seen in Figure 10 that, unlike the LNO:Dy, the ^4^F_9/2_ → ^6^H_15/2_ luminescence band of LSO:Dy at 295 K shows a structure with well-defined peaks. The most intense and narrow one is located near the long wavelength edge of the band at about 494 nm, whereas the other, slightly less intense neighbor is located at about 484 nm. These positions coincide with those of the most prominent lines in the luminescence spectrum of LSO:Dy at 10 K [35], pointing at the distribution of transition rates, rather uncommon in that the highest rates have transitions bridging the lowest crystal field component of the ^4^F_9/2_ with the highest energy crystal field components of the ^6^H_15/2_. Increasing population of higher energy components of the initial ^4^F_9/2_ multiplet counteracts this supremacy at higher temperatures, thereby changing the intensity distribution of the luminescence band components. The energy level schemes in Figure 11 are relevant to understanding the other peculiarities of the ^4^F_9/2_ → ^6^H_15/2_ luminescence bands shown in Figure 6, Figure 7, Figure 8, Figure 9 and Figure 10. In particular, the overall spectral width of the bands complies with an obvious rule that it is a sum of the energy spreads of the two levels involved in a transition. As a result, the overall bandwidth is the smallest for YAB:Dy, slightly bigger for LNO:Dy, and markedly larger for the remaining systems. Additionally, disparities of the spectral positions of the ^4^F_9/2_ → ^6^H_15/2_ luminescence bands in the crystals can be well understood. The same concerns the ^4^I_15/2_ → ^6^H_15/2_ luminescence band, except for in the GGAG:Dy sample, where only four out of eight crystal field components of the initial multiplet were located experimentally. It is worth noticing that energy separation between the lowest crystal field component of the ^4^I_15/2_ and the highest energy crystal field component of the ^4^F_9/2_ is systematically smaller than the ΔE_calc_ values.

The comments expressed above indicate that the rates of radiative transitions between thermalized luminescent multiplets and the terminal ground state of Dy^3+^ ions are other important structure-sensitive factors relevant to the termographic features of the systems under study. To get more close insight, we followed the theoretical approach employed in the past to interpret the optical temperature sensing of Er^3+^-doped calcium aluminate glass [47]. It was then proposed that the ratio of intensities I_ik_ and I_jk_ for a luminescence originated from a pair of thermally coupled levels can be calculated using the relation:

(4) $$\frac{I_{\mathrm{ik}}}{I_{\mathrm{jk}}}=\frac{c_{i}\left(\nu \right){\;A}_{\mathrm{ik}}{\;h\nu }_{\mathrm{ik}}{\;g}_{i}}{c_{j}\left(\nu \right)A_{\mathrm{jk}\;}{\;h\nu }_{\mathrm{jk}}{\;g}_{j}}\;\exp\left(-\frac{\Delta E}{\mathrm{kT}}\right)$$

where c(ν) denotes coefficients related to the spectral response of the instrument at luminescence wavelengths, hν denotes the energies of the emitted photons, A denotes the rates of radiative transitions related to the luminescence bands, g denotes the level of degeneracies, and ΔE denotes the energy separation between the two excited levels involved. The expression on the right-hand side of this general relation can be simplified when applied to Dy^3+^-doped systems, assuming c_i_(v) ≅ c_j_(v) and hν_ik_ ≅ hν_jk_, since the energy separation between the ^4^I_15/2_ and ^4^F_9/2_ multiplets is small. Next, employing the Judd–Ofelt approach, the values A_ik_ = A(^4^I_15/2_ → ^6^H_15/2_) and A_jk_ = A(^4^F_9/2_ → ^6^H_15/2_) can be determined from the relation [48]:

(5) $$A_{J^{\prime }J}=\frac{64\pi^{4}e^{2}}{3h\left(2J^{\prime }+1\right){\bar{\lambda }}^{3}}n{\left(\frac{n^{2}+2}{3n}\right)}^{2}\underset{t=2,4,6}{\sum }\Omega_{t}{\left|\langle \varphi_{a}\|U^{\left(t\right)}\|\varphi_{b}\rangle \right|}^{2}$$

where h is the Planck constant, $\bar{\lambda }$ is the mean wavelength of transition, n denotes the index of refraction, $\Omega_{t}$ are phenomenological intensity parameters, and ${\left|\langle \varphi_{a}\left\|U^{\left(t\right)}\right\|\varphi_{b}\rangle \right|}^{2}$ are doubly reduced matrix elements of unitary $U^{\left(t\right)}$ operators between the initial φ_a_ and terminal φ_b_ states. In this way, the rates A(^4^I_15/2_ → ^6^H_15/2_) and A(^4^F_9/2_ → ^6^H_15/2_) were calculated, inserting into Equation (5) the ${\left|\langle \varphi_{a}\left\|U^{\left(t\right)}\right\|\varphi_{b}\rangle \right|}^{2}$ values for Dy^3+^ taken from [49] and $\Omega_{t}$ parameters reported previously for systems under study. The calculated A(^4^I_15/2_ → ^6^H_15/2_)/A(^4^F_9/2_ → ^6^H_15/2_) ratios are compared in the last column of Table 5.

The incertitude of the data presented in the above tables and graphs is worth commenting on at this stage to ascertain the meaningfulness of the generalizations proposed in the following. Obviously, the incertitude of data for LNO:Dy is regarded as the highest because a strong thermally induced increase of optical absorption in the UV-blue gradually reduces the luminescence intensity, adversely affecting the incertitude of S_A_ and S_R_ values at higher temperatures. Reliability of the data for the remaining four systems is believed to be reasonable, i.e., the incertitude of the S_A_ and S_R_ values is assessed to be below 10%, and that of radiative transition rates derived from the Judd–Ofelt treatment is within 20%. To be safe with interpreting the results, we focused our attention on the S_R_ values at 350, and notice that these values for the YAB:Dy and LSO:Dy systems are higher than those for the LNO:Dy, GGAG:Dy, and GSO:Dy systems. In view of the gathered data, this finding cannot be attributed to the dissimilarity of structural factors consisting of the geometry and symmetry of Dy^3+^ sites, the number of non-equivalent Dy^3+^ sites, and the host anisotropy. A straightforward attribution involving the peculiarities of the crystal field splitting of the ^6^H_15/2_ ground state and the ^4^I_15/2_ and ^4^F_9/2_ excited multiplets or the nature and degree of spectral line broadening of Dy^3+^ transitions is not justified either. Instead, the S_R_ values mentioned above can be correlated meaningfully with R values gathered in the last column of Table 5. In fact, the R values for YAB:Dy and LSO:Dy are higher than those for GGAG:Dy and GSO:Dy, in agreement with the respective S_R_ values. For LNO:Dy, the R value is comparable to those for YAB:Dy and LSO:Dy. It disagrees with the rather low S_R_ value, likely because of the high incertitude of the luminescence data mentioned above.

At this stage, the correlation described above deserves some more detailed comments. The ratios R = A(^4^I_15/2_ → ^6^H_15/2_)/A(^4^F_9/2_ → ^6^H_15/2_) involve radiative transition rates A that follow from Equation (5). With simplifications resulting from a small energy difference between the ^4^I_15/2_ and ^4^F_9/2_ multiplets, we obtain:

(6) $$R=A{(}^{4}I_{15/2}\;\to {\;}^{6}H_{15/2})/A{(}^{4}F_{9/2}\;\to {\;}^{6}H_{15/2})\;\propto (0.0072\Omega_{2}+0.0003\Omega_{4}+0.0684\Omega_{6})/(0.0047\Omega_{4}+0.0295\Omega_{6})$$

Numbers preceding the Ω_t_ values in Equation (6) are values of matrix elements ${\left|\langle \varphi_{a}\left\|U^{\left(t\right)}\right\|\varphi_{b}\rangle \right|}^{2}$ of the unit tensor operators involved. It is worth noticing that the ${\left|\langle \varphi_{a}\|U^{\left(2\right)}\varphi_{b}\|\rangle \right|}^{2}$= 0 for the (^4^F_9/2_ → ^6^H_15/2_) transition. Therefore, Equation (6) predicts that the higher the Ω_2_ value is, the higher the R value will be, as seen in Table 5. The examination of Table 2 corroborates this prediction, revealing the increase of β from the lowest value for YAB:Dy to the highest value for GAGG:Dy. The correspondence between S_R_ values in Table 3 and calculated R values gathered in Table 5 is not rigorous, but it can be regarded as a general trend. It follows from experimental data and comments presented above that the dissimilarity of the rates of radiative transitions from crystal field levels induces thermal changes of inter-multiplet luminescent transition, which are not predicted by the Ω_t_ parameters determined from spectra at 300 K. Nevertheless, in our opinion, the predictions following from Equation (6) and from the luminescence branching ratio analysis may be useful during a preliminary assessment of the thermosensitive properties of new phosphor materials.

## 4. Conclusions

Detailed spectroscopic investigation of single crystal samples of Gd_2_SiO_5_:Dy^3+^, Lu_2_SiO_5_:Dy^3+^, LiNbO_3_:Dy^3+^, and Gd_3_Ga_3_Al_2_O_12_:Dy^3+^ fabricated by the Czochralski method and of YAl_3_(BO_3_)_4_:Dy^3+^ fabricated by the top-seeded high temperature solution method provided new and original information on their thermosensitive properties. Obtained results indicate that all of them are highly suitable for near room temperature sensing, with the relative thermal sensitivity S_R_ for YAl_3_(BO_3_)_4_:Dy^3+^ and Lu_2_SiO_5_:Dy^3+^ undoubtedly higher than those for the remaining systems studied. A thermally induced increase of absorption intensity for YAl_3_(BO_3_)_4_:Dy^3+^ due to color centers was inferred from the optical absorption spectra in the UV-blue region, recorded as a function of temperature between 295 K and 725 K. For LiNbO_3_:Dy^3+^, the thermally induced increase of absorption intensity, which we interpret in terms of temperature-dependent charge transfer (CT) transitions, is particularly strong, and hides absorption bands of Dy^3+^ in the UV-blue region above about 500 K, restricting, thereby, the thermal sensitivity region. The difference between thermosensitive features cannot be attributed directly to the dissimilarity of structural factors consisting of the geometry and symmetry of Dy^3+^ sites, the number of non-equivalent Dy^3+^ sites, and the host anisotropy. Based on the crystal field splitting of Dy^3+^ multiplets inferred from low temperature spectra, we interpret observed disagreement of the energy difference ΔE_calc_ obtained from the intensity ratio (FIR), fitting with ΔE_exp_ inferred from the centers of gravity of luminescence bands in terms of dissimilarity of rates of radiative transitions between individual crystal field levels. It was found that a meaningful correlation between the values of relative thermal sensitivity S_R_ and rates of radiative transitions of Dy^3+^ inferred from the Judd–Ofelt treatment exists. It was also concluded that the resulting predictions based on the Judd–Ofelt parameters and the luminescence branching ratio analysis may be useful during a preliminary assessment of the thermosensitive properties of new phosphor materials.

## Figures and Tables

**Table 1:** Selected structural and optical features of the crystals under study.

	Gd_3_Ga_3_Al_2_O_12_ (GGAG)	YAl_3_(BO_3_)_4_ (YAB)	LiNbO_3_ (LNO)	Gd_2_SiO_5_ (GSO)	Lu_2_SiO_5_ (LSO)
**Crystallographic system/** **space group**	Cubic/Ia-3d	Trigonal/R3c	Trigonal/rhombohedral/R3c	monoclinic/P2_1_/c	monoclinic/C2/c
**Unit cell (A)**	[31] A = 12.231	[32] a = 9.286 c = 7.231	[33] a = 5.15 c = 13.86	[34] a = 9.1105 b = 69783 c = 6.8544 β = 107.1411	[35] a = 14.277 (4) b = 6.6398 (4) c = 10.224 (6) β = 122.224 (1)°
**Cut-off phonon energy [cm^−1^]**	808	1200	630	900	900
**Bandgap (eV)**	5.9	5.7	4.2	5.95	5.95
**Dy^3+^ sites** **(Coordination No);** **Site symmetry**	Dy (CN = 8); D_2_	Dy (CN = 6); D_3_	Dy (CN = 6); Close to C_3_	Dy1 (CN = 9) Dy2 (CN = 7); C_3v_, C_s_	Dy1 (CN = 7) Dy2 (CN = 6); C_i_ for both sites

**Table 2:** Experimental luminescence branching ratios β of the Dy^3+ 4^F_9__/2_ → ^(2S+1)^L_J_ transitions and determined CIE values.

Luminescence Branching Ratios β in %										
	GAGG		YAB		LNO		GSO		LSO	
^4^F_9/2_ →										
^(2S+1)^L_J_	β_exp_		β_exp_		β_exp_		β_exp_		β_exp_	
^6^H_9/2_, ^6^F_11/2_	4		7		4		2		6	
^6^H_11/2_	8		11		9		5		5	
^6^H_13/2_	49		65		54		58		55	
^6^H_15/2_	42		17		33		35		34	
CIE	x	y	x	y	x	y	x	y	x	y
	0.393	0.396	0.405	0.456	0.431	0.426	0.403	0.412	0.401	0.432

**Table 3:** Comparison of relative sensitivity S_r_ determined for Dy-doped crystals.

Dy-Doped Material	Relative Sensitivity S_r_ [% K^−1^]	
	350 K	750 K
Gd_3_Ga_3_Al_2_O_5_ [this work]	0.93	0.23
YAl_3_(BO_3_)_4_ [this work]	1.18	0.24
LiNbO_3_ [this work]	0.92	0.37 (650 K)
Gd_2_SiO_5_ [this work]	0.77	0.23
Lu_2_SiO_5_ [this work]	1.73	0.23
Y_2_SiO_5_ [28]	-	0.41
GdVO_4_ [22]	1.80	-
Na_5_La_0.5_Dy_0.5_(WO_4_)_4_ [30]	1.80	0.30
NaDy(MoO_4_)_2_ [30]	0.75	0.38
YNbO_4_ [24]	1.40	0.36
Y_3_Al_5_O_12_ [28]	-	0.44
La_3_Ga_5.5_Ta_0.5_O_14_ [25]	1.47	0.34
BaYF_5_ [26]	1.10	0.25
Gd_2_Ti_2_O_7_ [27]	1.20	-
K_3_Y(PO_4_)_2_ [45]	1.31	-
Ba_3_Y_4_O_9_ [46]	1.34	-

**Table 4:** Calculated and experimental values of the energy gap ΔE between thermally coupled levels of Dy^3+^.

Crystal	ΔE_exp_ (350 K) [cm^−1^]	ΔE_exp_ (750 K) [cm^−1^]	ΔE_calc_ [cm^−1^]	ΔE_exp_ (350 K) − ΔE_calc_ [cm^−1^]	ΔE_exp_ (750K) − ΔE_calc_ [cm^−1^]
GAGG	1062	1158	945	-117	−213
YAB	1126	1116	916	-210	−200
LNO	1117	1161 *	1145	28	−16
GSO	1274	1212	934	−340	−278
LSO	1237	1261	839	-398	−422

* at 675 K for LNO:Dy.

**Table 5:** The Judd–Ofelt Ω_t_ parameters and calculated ratios R = A(^4^I_15/2_ → ^6^H_15/2_)/A(^4^F_9/2_ → ^6^H_15/2_).

System	Ω_2_ [10^−20^ cm^2^]	Ω_4_ [10^−20^ cm^2^]	Ω_6_ [10^−20^ cm^2^]	R
GAGG:Dy	1.33	4.12	3.02	2.00
YAB:Dy	10.04	2.04	2.31	2.97
LNO:Dy	5.42	1.14	2.51	2.66
LNO:Dy [50]	9.75	2.63	2.52	2.79
GSO:Dy	3.22	2.16	3.76	2.32
LSO:Dy	9.06	1.88	3.12	2.77
LSO:Dy (without hypersensitive transitions)	4.31	1.28	3.49	2.48

## Data Availability

The data presented in this study are available on request from the corresponding author.
